# Efficiency–Accuracy Trade-Off in Light Field Estimation with Cost Volume Construction and Aggregation

**DOI:** 10.3390/s24113583

**Published:** 2024-06-01

**Authors:** Bo Xiao, Stuart Perry, Xiujing Gao, Hongwu Huang

**Affiliations:** 1 State Key Laboratory of Advanced Design and Manufacturing for the Vehicle Body, Hunan University, Changsha 410012, China; xiaobo123@hnu.edu.cn; 2 School of Electrical and Data Engineering, University of Technology Sydney, Ultimo, NSW 2007, Australia; stuart.perry@uts.edu.au; 3 School of Smart Marine Science and Engineering, Fujian University of Technology, Fuzhou 350118, China; gaoxiujing@fjut.edu.cn; 4 Fujian Provincial Key Laboratory of Marine Smart Equipment, Fuzhou 350118, China

**Keywords:** depth estimation, light field, convolution neural network

## Abstract

The Rich spatial and angular information in light field images enables accurate depth estimation, which is a crucial aspect of environmental perception. However, the abundance of light field information also leads to high computational costs and memory pressure. Typically, selectively pruning some light field information can significantly improve computational efficiency but at the expense of reduced depth estimation accuracy in the pruned model, especially in low-texture regions and occluded areas where angular diversity is reduced. In this study, we propose a lightweight disparity estimation model that balances speed and accuracy and enhances depth estimation accuracy in textureless regions. We combined cost matching methods based on absolute difference and correlation to construct cost volumes, improving both accuracy and robustness. Additionally, we developed a multi-scale disparity cost fusion architecture, employing 3D convolutions and a UNet-like structure to handle matching costs at different depth scales. This method effectively integrates information across scales, utilizing the UNet structure for efficient fusion and completion of cost volumes, thus yielding more precise depth maps. Extensive testing shows that our method achieves computational efficiency on par with the most efficient existing methods, yet with double the accuracy. Moreover, our approach achieves comparable accuracy to the current highest-accuracy methods but with an order of magnitude improvement in computational performance.

## 1. Introduction

Light field imaging can serve as a significant potential tool for constructing 3D environments. Unlike traditional imaging, light field imaging captures a richer array of information, describing the distribution of light rays in three-dimensional space. Furthermore, it has applications in virtual reality [1,2], view synthesis [3], 3D reconstruction [4], and autonomous driving [5,6]. Depth estimation is a fundamental and critical step in these important research areas. However, these applications require not only high accuracy but also rapid generation speeds, thus necessitating that depth estimation processes simultaneously meet the demands for both estimation accuracy and computational efficiency.

In recent years, deep learning-based methods [6,7,8,9,10,11] have achieved significant advancements and demonstrated considerable potential in the realm of light field depth estimation. These approaches typically utilize all available light field image information, effectively improving the accuracy of depth estimation. However, the presence of abundant light field information in light fields leads to a substantial increase in network memory consumption and computational load. Therefore, many researchers have tried to improve computational speed by pruning redundant light field images, which indeed achieves good results. However, reducing the input information inevitably lowers accuracy and the perception of occlusion and texture regions. To address this issue, state-of-the-art methods [10] have utilized full correlation to construct matching cost volumes, replacing 3D convolution operations with 2D convolutions to further reduce computational load while incorporating multi-scale aggregation to boost accuracy. Nonetheless, full correlation tends to lose critical depth features and performs poorly in textureless areas. There are also many in-depth studies on the perception of textureless regions [12,13,14]. In summary, although 2D convolutions reduce data volume, they lose significant spatial depth information compared with 3D convolutions, rendering the network less effective in handling complex spatial scenes.

**Figure 1:**
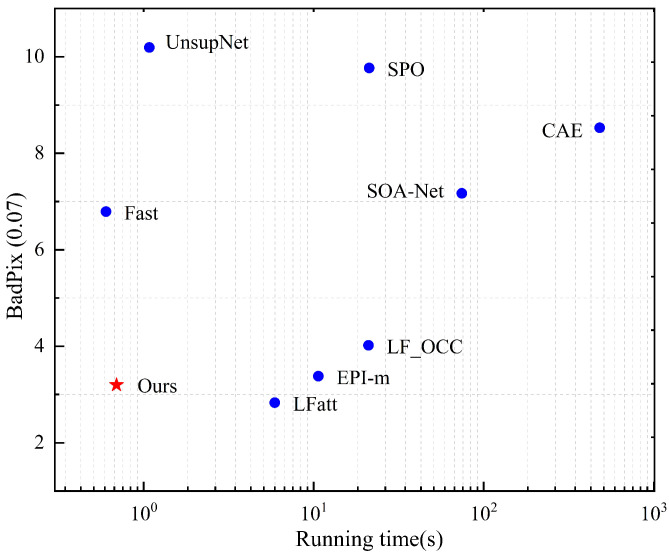
Comparison of efficiency and computation performance of light field disparity estimation algorithms.

To overcome these limitations, we propose a more effective method for cost volume construction and a cost aggregation architecture. Firstly, we replace full correlation with grouped correlation to enhance feature matching information. Secondly, we introduce feature dissimilarity operations to compensate for the shortcomings of feature correlation in textureless areas. Lastly, we present an architecture integrating Hourglass modules with 3D convolutions for multi-scale disparity fusion. This network structure more effectively captures multi-scale spatial features, and the use of 4D feature vectors further enhances the model’s ability to detect texture details, thereby improving both performance and accuracy in depth estimation. Our performance is shown in Figure 1.

## 2. Related Work

In this section, we review the main works in the direction of light field depth estimation based on traditional and deep learning methods.

### 2.1. Traditional Methods

Previous work on light field depth estimation has leveraged various light field attributes to obtain scene depth information. Traditional depth estimation methods generally fall into three categories: methods based on epipolar-plane images (EPIs) [15,16,17,18,19], methods based on multi-view stereo matching (MVS) [20,21,22,23], and methods based on refocusing [7,24,25,26,27].

The concept of EPIs was first introduced by Bolles et al., employing light field (LF) epipolar geometry to calculate line slopes for depth prediction. Wanner et al. [28] then incorporated it into light field depth estimation, using only horizontal- and vertical-direction EPIs of light field images, optimizing the results with a global consistency labeling algorithm. The EPI method significantly accelerated disparity estimation speed. Zhang et al. [29] further proposed the spinning parallelogram operator (SPO) to compute line slopes in EPIs, enhancing the accuracy of disparity estimation. Sheng et al. [30] used multi-directional EPIs to optimize slope estimation accuracy, achieving results surpassing the SPO. Heber et al. [21] developed a principal component analysis matching item for multi-view stereo reconstruction, combined with the projection of sub-aperture images (SAIs) for depth estimation. Jeon et al. [22] introduced a Fourier transform-based phase-shift theory to address small disparities between SAIs. In the refocusing approach, Tao et al. [24] combined defocus cues with consistency cues in light field images for depth map estimation, though performing poorly in occluded areas. Tao et al. [26] proposed a shadow-based refinement method to enhance the robustness of depth map estimation.

While these traditional methods have continuously progressed in accuracy and computational efficiency in light field disparity estimation, they are limited by nonlinear optimization and manually designed features. These features demand extensive computational resources and perform poorly in occluded and weakly textured areas, leaving substantial room for improvement in both accuracy and computational performance in disparity estimation.

### 2.2. Deep Learning Methods

In recent years, the use of deep convolutional neural networks (CNNs) for light field depth estimation has achieved impressive results. Focusing on disparity estimation accuracy, Tsai et al. [31] introduced an attention-based visual selection module that integrates the importance of each view with depth estimation, significantly enhancing robustness against noise interference. Building on this, Chen et al. [8] combined attention mechanisms with multi-level fusion networks, using fusion between different angular branches to further enhance disparity estimation accuracy. Most recently, Yang et al. [32] integrated local and global features within view feature cost volumes to address the challenges of occlusions and textureless regions, further improving disparity estimation accuracy. However, these methods, due to the use of redundant information and extensive 3D convolution operations, tend to be slower in generating disparity maps.

In another direction, Heber et al. [33] proposed a U-shaped artificial neural network to extract geometric information from light fields for depth estimation, initially utilizing EPIs. Subsequently, Shin et al. [34] used CNNs to extract geometric disparities from EPIs and proposed a fully convolutional end-to-end network. Further, Huang [10] designed a disparity estimation model that replaced 3D convolutions with 2D convolutions, significantly reducing the learning parameters and enhancing computational performance. These methods, which generate disparity maps using a lower proportion of light field images as input, have good computational performance but are limited in disparity estimation accuracy and robustness against real-world noise, especially compared with methods that use inputs from all views.

Finally, previous research has already shown the advantages of deep neural networks in light field depth estimation. However, there has been insufficient focus on balancing accuracy and computational performance, often leading to a trade-off when generating disparity images. In this paper, we propose a lightweight convolutional neural network that employs multi-disparity cost aggregation. This network extracts richer depth information from fewer input data and achieves a balance between computational load and depth estimation accuracy.

## 3. Method

**Figure 2:**
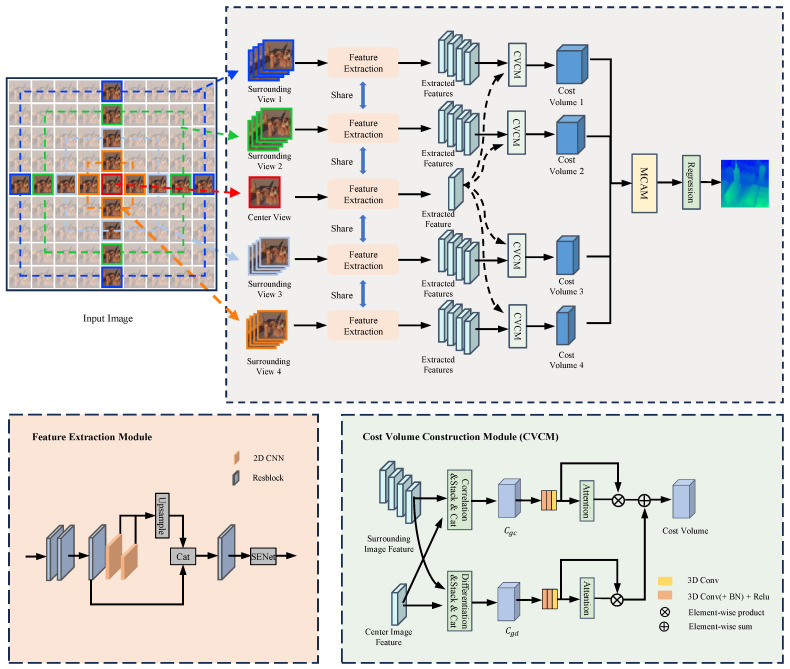
An overview of our network. The term “MCAM” denotes the multi-view cost volume aggregation module, and its specific structure is detailed in Figure 3.

In this paper, we introduce a novel method that balances efficiency and accuracy in light field depth estimation. The overall architecture of the network is depicted in Figure 2. Initially, acknowledging the redundancy in light field images, we only use sub-aperture images (SAIs) from the horizontal and vertical directions as inputs to reduce computational costs as much as possible. A shared feature extraction module is then employed to extract SAI features (Section 3.1). Following this, we construct cost volumes based on the features of surrounding pixels after pixel shifting and central features. In this process, we propose a hybrid cost volume network to enhance detail perception (Section 3.2). Finally, a multi-scale disparity cost aggregation module is used to synthesize mixed cost depth information, which is then processed by a disparity regression module to predict the disparity map (Section 3.3).

### 3.1. Feature Extraction

The extraction of effective features is crucial to estimating the disparity map, particularly due to the small disparity range in light fields, which complicates accurate estimation in low-texture and occluded areas. To address this, we use multiple basic residual blocks for preliminary feature extraction, applying stride-2 convolutions for downsampling. The feature maps are downsampled at two different scales and subsequently restored to their original scale through bilinear interpolation. These features at different levels are concatenated and fused via convolution, and an SENet [35] module is added to enhance the weighting of key feature channels. The resultant feature map serves as the input to our hybrid cost volume network.

### 3.2. Texture-Aware Cost Volume

After extracting features of SAIs, a 4D cost volume is constructed to predict the disparity map by establishing a correspondence between shifted surrounding view features and the central view feature. We use parallel plane parameterization to represent the four-dimensional light field, L(x,u), where x and u are the spatial and angular coordinates, respectively. $I_{c}\left(x,u_{c}\right)$ is the central view. The disparity of the light field is denoted by $d(x,u_{c})$, and $u_{c}$ denotes the center view position. According to LF geometry, given a surrounding SAI $I_{s}\left(x,u\right)$, the reconstructed central view, ${\tilde{I}}_{c}\left(x,u\right)$, can be expressed as

(1) $$\begin{array}{c} {\tilde{I}}_{c}\left(x,u\to u_{c}\right)=I_{s}\left(x+\left(u-u_{c}\right)\cdot d\left(x,u_{c}\right),u\right) \end{array}$$

By using this equation, we can reconstruct the central view, ${\tilde{I}}_{c}\left(x,u\right)$, from the surrounding views, $I_{s}\left(x,u\right)$, where $x+\left(u-u_{c}\right)\cdot d\left(x,u_{c}\right)$ calculates the displacement caused by the disparity.

Typically, the full correlation cost volume is obtained by using correlation operations [10,36] between the distorted features of surrounding views and the central view to regress the disparity map. However, relying solely on full correlation can result in the loss of significant information. To further reduce the computational load, we group features to compress the matching cost volume. The number of channels of a univariate feature is denoted by $N_{c}$, and the channels are uniformly divided into $N_{g}$ groups along the channel dimension. Therefore, each group feature has $N_{c}/N_{g}$ channels. Correlation is then computed for each group. The correlation between the surrounding view features, $F_{s}^{g}$, and the central view features, $F_{c}^{g}$, is represented as follows:

(2) $$\begin{array}{c} C_{gc}\left(d,x,g\right)=\frac{N_{g}}{N_{c}}\langle F_{c}^{g}\left(x,u_{c}\right),F_{s}^{g}\left(x+(u-u_{c})\cdot d,u\right)\rangle \end{array}$$

where $\langle \cdot ,\cdot \rangle$ represents the inner product of two features and $C_{gc}$ is the correlation cost volume for feature group *g* and disparity *d*.

However, due to the low values of features in textureless areas, the multiplication operation in the correlation process results in a small variance between the feature costs at the correct and incorrect depths. This can easily lead to interference by noise and incorrect depth estimation. To address this issue, we introduce a new set of cost volumes. We construct cost volumes by using the sum of absolute differences between feature views, effectively increasing the variance range of depth-related feature costs and enhancing the network’s perception of the correct depth in textureless areas. The differentiation cost volume between feature view pixels is represented as follows:

(3) $$\begin{array}{c} C_{gd}\left(d,x,g\right)=\sum_{i=1}^{N_{c}/N_{g}}\left|F_{c}^{g}\left(x,u_{c}\right)-F_{s}^{g}\left(x+(u-u_{c})\cdot d,u\right)\right| \end{array}$$

Furthermore, the cost volumes are stacked in the depth direction as a 4D array (G×D×H×W) and then concatenated to connect volumes with the same disparity scale, forming the initial cost volume (4G×D×H×W). Here, *G* represents the number of groups, *D* the number of disparity layers, and *H* and *W* the dimensions of the input image. It is important to note that the relationship of disparity scales at different distance angles is proportional to the distance (*d*). Here, we define the maximum disparity for the innermost view as $d_{max}$, and considering that disparity estimation requires multiple downsampling operations, we set the total number of disparity layers to be even, with a disparity range of $\left[-d_{max},1+d_{max}\right]$ and disparity levels set to $2+2d_{max}$. Similarly, the disparity for the outermost view is set to $\left[-4d_{max},1+4d_{max}\right]$, with disparity levels set to $2+4d_{max}$. The disparity level refers to the number of discrete disparities within the interval from the minimum to the maximum disparity.

Finally, through the network we propose, the correlation cost volume and the differentiation cost volume are fused, as illustrated in the CVCM module shown in the lower right corner of Figure 2. The correlation cost volume and the differentiation cost volume are processed separately through 3D convolution and a 3D channel attention mechanism to extract matching information. They are then combined to form the final cost volume.

During the construction of the cost volume matching process, we employed a grouping method to compress the most memory-intensive part, reducing computational load and memory consumption. Additionally, we proposed a correlation and dissimilarity fusion structure to enhance perception and accuracy in textureless regions.

### 3.3. Multi-View Cost Volume Aggregation and Disparity Regression

**Figure 3:**
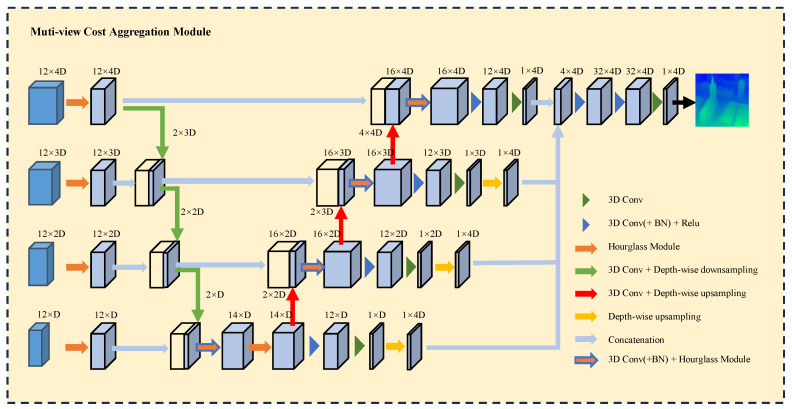
Multi-View Cost Aggregation Module architecture. Note that the feature size annotations in our diagram omit the dimensions (H×W) of the input light field images.

To fuse cost volumes of different scales for disparity estimation and enhance the model’s performance in occluded areas, we propose a multi-level fusion strategy. Given that spatially occluded areas in the central view are visible from other directional viewpoints, we employ a structure similar to U-Net, featuring upsampling and downsampling, along with Hourglass modules, to extract useful features from unoccluded areas. Furthermore, to achieve better accuracy in depth estimation, we designed a multi-level scale disparity fusion structure to enhance feature robustness. As shown in Figure 3, the first layer input is the maximum disparity cost volume, capturing the broadest spatial information to provide a comprehensive initial perspective for depth estimation. Information from different disparity scales is integrated from largest to smallest, offering a multi-level feature fusion strategy that transitions from local to global and back to local. Finally, each layer’s disparity is upsampled to the same size and fused by using three-dimensional convolution.

**Figure 4:**
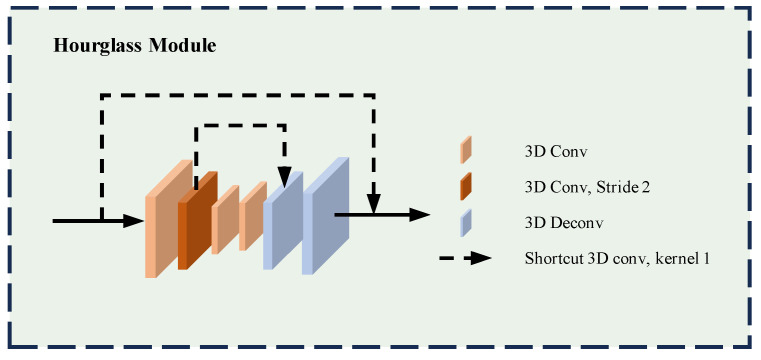
Hourglass module structure.

Furthermore, to more effectively capture global and local texture features at the current scale of each layer, we have embedded Hourglass modules [37,38] at every level. These modules not only recognize large-scale structures within the feature cost volumes but also process fine textures and edge details. The specific details of this process are illustrated in Figure 4. This approach ensures a more nuanced and comprehensive analysis of the light field data, significantly enhancing depth estimation accuracy and detail.

After obtaining the final cost volume, each pixel is represented by a vector of length $D_{max}$, containing the probabilities of all disparity levels. We use the softmax activation function introduced in [39] to generate a continuous distribution of disparity predictions. The predicted disparity value, $\hat{d}$, is defined as

(4) $$\begin{array}{c} \hat{d}=\sum_{d=D_{min}}^{D_{max}}d_{i}\times \mathrm{softmax}\left(-C_{f}\right) \end{array}$$

where $\hat{d}$ represents the predicted disparity by the pixel; $D_{min}$ and $D_{max}$ denote the minimum and maximum disparities of the outermost view, respectively; and $C_{f}$ is the predicted cost at disparity $d_{i}$.

## 4. Experiments

In this section, we will introduce the details and results of our implementation. Finally, we will compare our method with several state-of-the-art light field depth estimation methods.

### 4.1. Dataset and Implementation Details

The 4D light field dataset [40] is widely regarded as a benchmark for evaluating light field image disparity estimation methods. This dataset, rendered by using Blender, includes 28 densely arranged synthetic light field scenes and their corresponding ground-truth disparity maps divided into four subsets: “Stratified”, “Test”, “Training”, and “Additional”. These scenes incorporate a mix of various materials, lighting conditions, and complex spatial occlusions. All light field data possess a 9 × 9 angular resolution and a 512 × 512 image resolution.

We utilized the “Additional” category of the dataset for training our model. During training, we randomly cropped the SAIs into 48 × 48 grayscale patches and applied various image augmentation techniques, including random rotation, brightness and contrast adjustments, and noise injection. Inspired by Zhao [41], we adopted a joint L1 and SSIM [42] loss function for our training network, denoted by loss term *L*, and optimized it by using the Adam method [43]. The loss function is formulated as follows:

(5) $$\begin{array}{c} L=\frac{1}{M}\sum_{i,j}\left(\alpha \frac{1-\mathrm{SSIM}(d_{i,j},{\tilde{d}}_{i,j})}{2}+\left(1-\alpha \right)∥d_{i,j}-{\tilde{d}}_{i,j}∥\right) \end{array}$$

where $\tilde{d}$ represents the predicted disparity map, *d* is the true disparity map, and *M* denotes the number of pixels, with α being set to 0.9. We tested weights α ranging from 0.1 to 0.9 and found that as the weight increases, accuracy in depth estimation also improves. The proposed method was implemented with PyTorch platform [44] and optimized by using the Adam [43] ($\beta_{1}=0.9$, $\beta_{2}=0.999$) optimizer. The batch size was set to 16, and the initial learning rate was $10^{-3}$, decaying by 0.8 every 100 epochs. The total training comprised 1000 iterations. Our model was trained on a PC equipped with an Nvidia 6000× GPU, requiring approximately three days.

### 4.2. Comparison to State-of-the-Art Methods

We compared our approach to various state-of-the-art methods, including traditional methods [25,29,45], unsupervised deep learning methods [46,47], and supervised deep learning methods [10,31,34,48].

To demonstrate the accuracy of our method, we compared its performance with other state-of-the-art methods on the “Stratified” and “Training” categories of 4D LF data in terms of bad pixel rate (BadPix) (0.07) and Mean Squared Error (MSE). The comparative results are reported in Table 1, showing that our method achieved good results overall compared with the other methods.

For performance comparison, to ensure fairness, we executed these methods on the same platform and compared the average running time for these scenes. The results, as shown in Table 2, indicate that our method outperforms the other methods, except for the Fast method. Additionally, while our method’s accuracy is second only to LFAtt [31], it computes faster. The traditional methods CAE [25] and SPO [29] were run on a CPU platform configured with an Intel i7-10850H.

**Figure 5:**
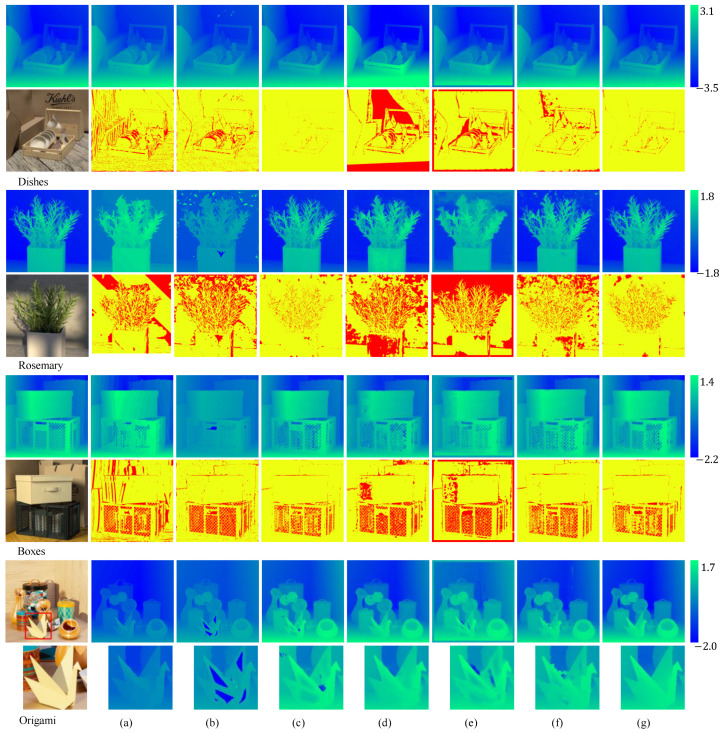
Quantitative comparison of the performance of different methods on the HCI light field benchmark. (**a**–**g**) show the results for CAE [25], SPO [29], LFAtt [31], Fast [10], Distrib [46], EPI-m [34], and our method, respectively. The first row in each scene represents the estimated disparity corresponding to the original image, and the second row displays the distribution of bad pixels, with red indicating areas where the bad pixel rate exceeds 0.07.

In addition, we also subjectively compared the performance of our method with other methods in textureless and occluded areas. Figure 5 displays visual comparison results across four scenarios: “Dish”, “Dots”, “Rosemary”, and “Origami”. The depth estimation results and errors for the first three scenes show that our method performs well in spatial areas with occluded edges, comparable to the best methods available [31]. Additionally, in the “Origami” scene, as indicated, our method achieves better accuracy in the marked textureless areas. Overall, the results demonstrate that our method exhibits superior performance and robustness in handling the challenge of textureless and occluded areas.

**Figure 6:**
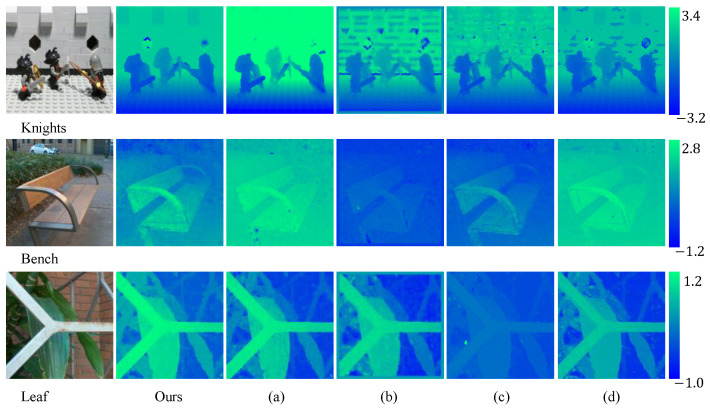
Qualitative results of real-world light field images. (**a**–**d**) represent depth maps generated by deep learning-based methods Fast [10], Distrib [46], EPI-m [34], and LFAtt [31], respectively.

To comprehensively evaluate the performance of our method, we also used real-world datasets for testing and comparison with state-of-the-art methods. As illustrated in Figure 6, the depth maps generated by our method are more consistent and exhibit less noise. This indicates that our approach can be effectively generalized to real LF depth estimation. The scenes “Bench” and “Leaf” were captured by using our Lytro Illum camera, and “Knights” was captured by using the gantry setup from the Stanford Light Field Archive.

### 4.3. Ablation Study

We conducted extensive ablation experiments to analyze the effectiveness of our method. Our ablation study includes the trade-off between performance and efficiency, the choice of disparity cost operations, and the combination of loss functions.

#### 4.3.1. Disparity Cost Calculation

**Figure 7:**
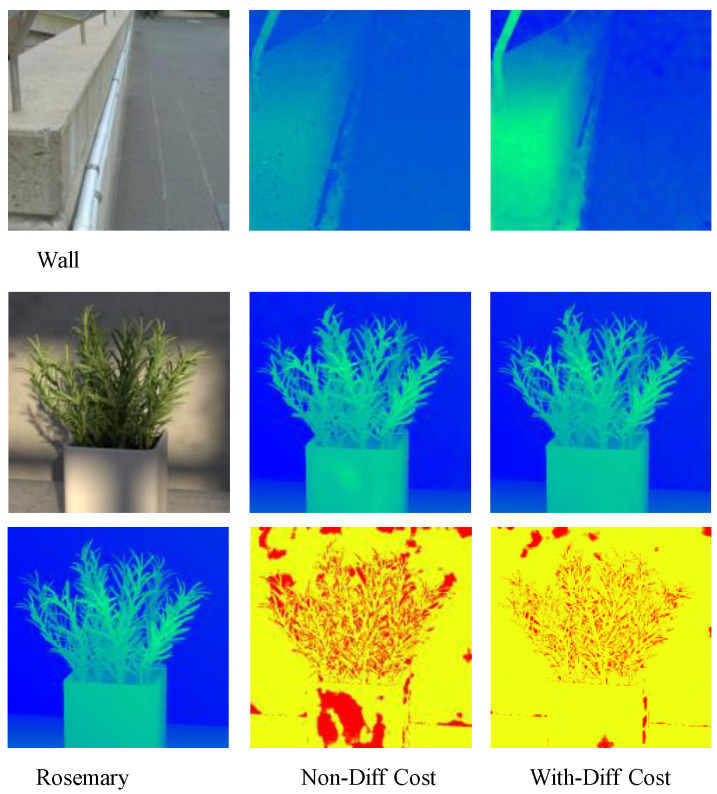
Disparity maps in synthetic and real scenes. “Wall” represents a light field image captured by us, and “Rosemary” is the synthetic light field image.

The disparity cost has a significant impact on accuracy in depth estimation, so it is crucial to choose the appropriate cost generation operations. We conducted separate tests with and without the feature dissimilarity operation on the HCI 4D LF benchmark. When we removed the feature dissimilarity operation, the network’s performance in terms of both MSE and BadPix (0.07) deteriorated. Figure 7 illustrates the influence of feature dissimilarity on depth estimation within the aggregated cost volume. It indicates that adding feature dissimilarity effectively improves the network’s performance in weak-texture regions and enhances its robustness.

#### 4.3.2. Computational Cost

To validate the impact of the light field input distribution and the number of grouped aggregation channels on the computational performance and effectiveness of our network, we used three different combinations of horizontal, vertical, and diagonal distributions as input variables for the network, as well as varying numbers of cost aggregation groups as intermediate variables. The results are shown in Table 3.

The number of image inputs has a significant impact on network performance. Increasing the data based on the distribution of horizontal and vertical directions results in a small gain in accuracy but a significant performance drop. As indicated in the fourth column of the table, the optimal number of grouped aggregation channels is four, and the number of groups has little impact on network performance. Overall, our method achieves optimal performance in the network with the choice of input data distribution and the number of groups.

#### 4.3.3. Effectiveness of Cost Aggregation Network

To verify the role of the cost aggregation network within the overall architecture, we constructed a cost aggregation network by using the Resnet structure [50], as depicted in Figure 4, as a benchmark module for comparison. Considering memory limitations, the input cost volumes were uniformly resampled to the same dimensions B×12×2D×H×W before aggregation, followed by two 3D convolutions and eight 3D convolutions within the Resnet structure, and then depth regression. We trained for a total of 500 epochs. The results on the HCI light field benchmark dataset are shown in Table 4.

The depth estimation accuracy achieved by our cost aggregation network structure is higher than that of the comparison experiment, indicating that our proposed multi-scale cost aggregation module plays a significant role in improving accuracy.

Overall, our ablation study validates the approach we proposed, demonstrating that each modular component of our model makes a valuable contribution to the final results.

## 5. Discussions and Conclusions

Despite achieving commendable results in both disparity estimation accuracy and computational performance, our method still has certain limitations. First, our approach heavily relies on high-quality light field data, and its robustness to distortion and noise in light field images is weak, especially when the number of input data is limited. Consequently, the performance of our method might decrease when applied to real-world light field data. In the future, we could explore integrating specially designed network modules to mitigate the impact of distortion.

Secondly, our ablation studies reveal that the total number of input data significantly impacts computational performance. While optimizing the cost aggregation can reduce the number of parameters and thus computation time, it does not substantially affect overall performance. Simultaneously, the quality of cost volume construction directly influences accuracy in depth estimation in challenging areas. Future work could, therefore, focus on exploring better input structures and cost construction methods to balance computational performance and accuracy.

In this paper, we propose an end-to-end network architecture that trades off computational performance and depth estimation accuracy. Our feature dissimilarity cost construction method effectively compensates for the shortcomings of feature correlation, enhancing network accuracy in textureless areas. Moreover, our multi-scale cost aggregation architecture significantly improves depth estimation accuracy while maintaining good computational performance. Overall, compared with state-of-the-art methods, our approach achieves the best trade-off between computational performance and accuracy, as demonstrated on a broad HCI benchmark set and on a real-world light field dataset.

## Figures and Tables

**Table 1:** Quantitative comparison with other state-of-the-art methods in terms of BadPix (0.07) and MSE on 4D light field benchmark dataset [40]. Lower scores represent better performance.

Methods	CAE [25]		SPO [29]		LFAtt [31]		Fast [10]		Distrib [46]		EPI-m [34]		Ours	
	BP07	MSE	BP07	MSE	BP07	MSE	BP07	MSE	BP07	MSE	BP07	MSE	BP07	MSE
Backgammon	2.967	5.170	2.608	3.607	3.126	3.648	3.323	1.818	19.22	13.68	2.229	2.579	2.342	1.830
Boxes	19.86	10.01	15.98	12.19	11.04	3.996	18.28	4.532	28.7	15.92	12.34	5.968	10.50	3.786
Cotton	3.562	1.844	2.343	2.009	0.272	0.209	0.984	0.341	21.22	12.74	0.55	0.287	0.537	0.265
Dino	5.752	0.407	2.469	0.407	0.848	0.093	3.122	0.208	13.41	8.775	1.207	0.157	1.245	0.125
Dots	15.50	8.127	35.29	16.68	1.432	1.425	16.36	3.524	35.70	6.663	2.490	1.475	3.939	2.322
Pyramids	1.822	0.053	0.271	0.02	0.195	0.004	0.407	0.017	8.992	2.029	0.159	0.008	0.382	0.008
Sideboard	11.05	0.876	7.670	1.027	2.870	0.530	7.472	0.823	19.81	11.31	4.462	0.798	3.865	0.584
Stripes	8.534	3.268	11.59	6.276	2.933	0.892	4.125	0.192	30.17	2.91	2.457	0.932	2.812	0.350

Bad pixel ratio of 0.07 (BP07) and MSE (multiplied with 100) are the metrics for accuracy evaluation, where lower scores represent better performance. The best result is shown in deep blue and the second best in orange.

**Table 2:** Quantitative comparison of the average performance and efficiency with state-of-the-art methods on the 4D LF Benchmark.

Method	Average BadPix (0.07)	Average Running Time/s
CAE [25]	8.530	481.0
SPO [29]	9.770	21.21
LFAtt [31]	2.836	5.913
Fast [10]	6.792	0.601
Distrib [46]	22.15	4.830
EPI-m [34]	3.383	10.65
SOA-Net [48]	7.170	74.30
LF_OCC [45]	4.021	21.00
UnsuperNet [47]	10.19	1.079
Ours	3.203	0.693

The data for methods SOA-Net [48] and UnsuperNet [47] are cited from reference [49]. The best result is shown in deep blue and the second best in orange.

**Table 3:** Quantitative comparison of results with different inputs and varying numbers of aggregation group channels.

Input			Number within Group	Average MSE (×100)	Average BidPix (0.07)	Time (s)
Horizontal	Vertical	Diagonals				
√			4	2.552	8.540	0.410
		√	4	4.445	10.132	0.673
√	√	√	4	1.205	3.102	1.612
√	√		**4**	**1.159**	**3.203**	**0.693**
√	√		8	1.275	3.560	0.740
√	√		1	2.148	4.169	0.613

Horizontal, Vertical, and Diagonals represent inputs at $0^{\circ }$, $90^{\circ }$, and two diagonal directions through the central subspace image in the light field array, respectively.

**Table 4:** Depth estimation results of cost aggregation module and resnet benchmark module on HCI dataset.

	Average MSE	Average BP (0.07)
ResNet-based	3.201	7.422
Ours	**1.456**	**4.232**

## Data Availability

The data presented in this study are available upon request from the corresponding author. The data are not publicly available due to the protection of intellectual property.
